# Evaluation in primary health care in Brazil: reflections on institutionalization

**DOI:** 10.1590/0102-311XEN182025

**Published:** 2026-11-09

**Authors:** Eronildo Felisberto, Gabriella de Almeida Raschke Medeiros, Isabella Samico, Zulmira Maria de Araújo Hartz, Luciana Santos Dubeux, Ana Coelho de Albuquerque, Juliana Martins Barbosa da Silva Costa

**Affiliations:** 1 Grupo de Estudos em Gestão e Avaliação em Saúde, Instituto de Medicina Integral Prof. Fernando Figueira, Recife, Brasil.; 2 Secretaria de Atenção Primária à Saúde, Ministério da Saúde, Brasília, Brasil.; 3 Centro Acadêmico do Agreste, Universidade Federal de Pernambuco, Caruaru, Brasil.

**Keywords:** Health Evaluation, Primary Health Care, Sustainable Development Goals, Institucionalization, Health Policy, Evaluación en Salud, Atención Primaria de Salud, Objetivos del Desarrollo Sostenible, Institucionalización, Política de Salud

## Abstract

This article presents reflections on the institutionalization of health evaluation. By systematizing developments over the last twenty-five years in the field of evaluation in primary health care (PHC) in Brazil, it examines them in light of theoretical approaches, seeking to understand the processes of change shaped by movements that have influenced both practices and the use of evaluation. Recognizing that institutionalizing evaluation requires a systemic approach, the article highlights the impact of these changes on the thinking, actions and interactions of those involved, as well as on organizational practices and decisions. Evaluation in PHC should be established as a strategic, ethical and dialogical practice aligned with the democratic commitments of the Brazilian Unified National Health System (SUS) and the challenges posed by contemporary social transformations. Drawing on theoretical conceptions of planetary health and transformative evaluation, the article argues that evaluation should intentionally serve as a driver of social transformation and as a space for qualified listening, the visibility of vulnerabilities, the promotion of equity and intersectoral coordination. It further suggests that institutionalization should extend beyond the formal arrangements of health systems to include strategies for embedding evaluation in territories and in the relationships between the state and society. The aim is to stimulate debate by proposing pathways through which the institutionalization of PHC evaluation may achieve broader legitimacy and become entrenched as part of public service ethics and social responsiveness.

## Abstract (es)

Este artículo presenta reflexiones sobre la institucionalización de la evaluación en salud. Al sistematizar eventos ocurridos en los últimos 25 años en el ámbito de la evaluación de la atención primaria de salud (APS) en Brasil, este estudio los contextualiza dentro de enfoques teóricos, buscando comprender los procesos de cambio resultantes de los movimientos que moldearon las prácticas y el uso de la evaluación. Considerando que la institucionalización de la evaluación requiere un enfoque sistémico, se destaca la influencia de estos cambios en el pensamiento, acciones e interacciones de los involucrados, así como en las prácticas y decisiones organizativas. La evaluación en la APS debe establecerse como una práctica estratégica, ética y dialógica, alineada con los compromisos democráticos del Sistema Único de Salud brasileño (SUS) y los desafíos de las transformaciones sociales contemporáneas. Inspirado en conceptos teóricos de salud planetaria y evaluación transformadora, argumenta que la evaluación debe asumir intencionalmente un papel de transformación social y un espacio para la escucha cualificada, la visibilidad de las vulnerabilidades, la promoción de la equidad y la coordinación intersectorial. Sugiere que su institucionalización no debe limitarse a los arreglos formales de los sistemas de salud y que debe considerar estrategias para arraigarla en los territorios y en las relaciones entre el Estado y la sociedad. Su objetivo es animar el debate al proponer pistas sobre cómo la institucionalización de la evaluación en la APS puede obtener una legitimidad más amplia y consolidarse como parte de la ética del servicio público y de la responsabilidad social.

An article published in 2024 ^1^ by researchers from the PHC Research Network of the Brazilian Association of Public Health (Rede APS/ABRASCO, acronym in Portuguese), paved the way for a necessary reflection on the directions of evaluation in primary health care (PHC) in Brazil. The text presents a historical outline of monitoring and evaluation initiatives in PHC, proposes a research agenda committed to strengthening the Family Health Strategy (FHS) and consolidating the Brazilian Unified National Health System (SUS, acronym in Portuguese), and advocates the development of a Brazilian National Monitoring and Evaluation Policy to guide PHC management and care within the SUS.

To advance this reflection, this article aims to develop a historical-critical analysis of the institutionalization of evaluation in PHC in Brazil, combined with a theoretical reflection on its foundations, tensions and challenges in light of contemporary social and territorial transformations. To this end, it adopts an intentional, institutional and political selection of relevant events, focusing on those that, over the last twenty-five years, have shaped the field of Monitoring and Evaluation of PHC in Brazil (M&E/PHC), based on the model proposed by the Brazilian Ministry of Health between 2003 and 2006 for structuring an evaluation policy for PHC ^2^.

## Trajectory and mechanisms of PHC evaluation in Brazil

Two important initiatives preceded this period. The Primary Health Care Information System (SIAB, acronym in Portuguese), created in 1998 as a managerial tool to monitor the activities and results of the FHS teams, incorporated features such as the micro-spatialization of data, the generation of indicators focused on the cycle of organization of activities, and the availability of information at levels of aggregation, which contributed to the debate on health accountability ^3^. In the same year, the Primary Health Care Indicators Pact (PIAB, acronym in Portuguese) regulated the monitoring and evaluation process based on indicators, flows and deadlines, influencing financial transfers to municipalities. It also defined the role of each management level in tripartite negotiations and the establishment of commitments in light of the Basic Operational Norms ^4^.

In subsequent years, the FHS expanded significantly and consolidated its central role in shaping PHC. This process required a new institutional configuration within the Brazilian Ministry of Health. In the 2000s, the Department of Primary Care and the Monitoring and Evaluation Unit were created. The strengthening of this institutional structure enabled the development of M&E processes, encouraged evaluative research and fostered collaboration with educational institutions. These initiatives aimed to provide professional capacity building and ensure financial resources for the evaluation of the FHS within the scope of the Family Health Expansion and Consolidation Project (PROESF, acronym in Portuguese) ^5^.

During 2003-2006, significant progress was observed in M&E/PHC, with strategies aimed at its institutionalization. This process was driven by the proposal of a sectoral evaluation policy, based on an expanded debate within the Brazilian Ministry of Health and the Tripartite Inter-Managerial Commission, which defined some guidelines: partnerships with academic institutions and professional associations; mobilization of institutional actors; identification of funding sources; and the definition of strategies for capacity building in evaluation ^2^ ^,^ ^6^ ^,^ ^7^.

These guidelines informed coordinated strategies aimed at disseminating and fostering an evaluative culture within PHC: reformulating the SIAB and making its data publicly available; revitalizing the PIAB negotiation process; producing, analyzing and disseminating indicators from other information systems; developing tools to facilitate the use of information; encouraging and managing research; publicizing successful experiences; training staff; and raising funds for evaluation ^2^ ^,^ ^6^.

As a result, innovative projects sought to overcome evaluative practices restricted to interventions: Strengthening Technical Capacity in M&E in State Health Departments (2004), implemented through technical cooperation with collaborating centers; the PROESF Baseline Studies (2005), which prioritized national research institutions with independent monitoring by Abrasco; and the Evaluation for Quality Improvement (EQI) (2005), a self-assessment instrument directed at the FHS. Institutional support for the creation of the Technical Group for Monitoring and Evaluation of Health Programs and Policies (GT-Avaliação, acronym in Portuguese) of Abrasco should also be highlighted, as it encouraged cooperation between academic institutions and health services ^8^ ^,^ ^9^ ^,^ ^10^ ^,^ ^11^ ^,^ ^12^. These initiatives formed part of the Brazilian National Policy for Monitoring and Evaluation of Primary Care (PNMA/AB, acronym in Portuguese), which functioned “...*as an institutional standard to promote the development of an evaluative culture, among others that guided evaluative practice in primary care in Brazil...*” ^6^ (p. 38), such as the Pact for Health (2004) and the Brazilian National Primary Health Care Policy (PNAB, acronym in Portuguese) (2006) ^6^ ^,^ ^9^ ^,^ ^12^.

The milestones mentioned above defined guidelines and responsibilities for M&E/PHC. However, federal initiatives during the period 2007-2010 were limited to a sample survey on the situation of PHC in Brazil, support for the Rede APS and the actions defined for the second phase of PROESF. It was only from 2011 onward that new developments toward the institutionalization of evaluation emerged, with the establishment of the Improvement Access and Quality National Program of Primary Health Care (PMAQ-AB, acronym in Portuguese) ^12^ ^,^ ^13^.

This program encouraged the revision of the EQI, resulting in the document *Self-Assessment for Improving Access and Quality in Primary Care* [*Autoavaliação para Melhoria do Acesso e da Qualidade da Atenção Básica - AMAQ*] ^14^ which expanded the possibility of applying quality standards to other models of care beyond the FHS. The PMAQ proposed greater public accountability, continuous quality improvement and recognition of team performance, incorporating mechanisms for evaluation, target-setting agreements and performance-linked financial transfers. This represented a turning point in the policy of PHC funding and management ^12^ ^,^ ^13^ ^,^ ^15^ ^,^ ^16^.

The PMAQ sought to integrate evaluation and funding by interconnecting the quantitative and qualitative dimensions of care. Its framework included: voluntary adherence by municipalities; target-setting agreements between teams and management; team self-assessment; continuous monitoring; external evaluation; performance-based certification; and financial incentives. This repositioned evaluation as a central instrument in PHC management, incorporating it as a driver of transformation and emphasizing its formative and strategic role. PHC could now benefit from a continuous process of data production to support local planning, strengthening institutional capacity by encouraging the use of evaluation by managers and professionals at different levels, in addition to incorporating external evaluation by research institutions ^15^ ^,^ ^16^.

In 2013, the Brazilian Ministry of Health established the e-SUS Primary Care system and the Citizen’s Electronic Health Record. These initiatives aimed to overcome the limitations of the SIAB by enabling both the recording of service delivery and the longitudinal monitoring of care. The scope of available data was expanded, promoting interoperability among systems and making a significant contribution to M&E/PHC ^1^ ^,^ ^13^ ^,^ ^15^ ^,^ ^16^.

Despite these advances, another shift is observed in PHC from 2016 onward. The adoption of political guidelines contrary to SUS principles intensified institutional movements to dismantle and discredit initiatives such as the PMAQ. In 2019, the Previne Brasil program was established as the new PHC funding model, linking financial transfers to municipalities to criteria such as weighted capitation based on the registered population, performance-based payments according to production indicators and incentives for strategic actions, disregarding the constitutional principle of universal access to health care ^1^ ^,^ ^17^.

In 2024, with the resumption of democratic processes and the reorganization of federal co-funding, the Brazilian Ministry of Health readjusted these mechanisms in line with SUS principles, establishing a quality component with indicators agreed upon through a tripartite process ^18^. From the perspective of evaluation, the new model reintroduces challenges already observed in the PMAQ experience, particularly regarding the central role played by indicators, the induction of behaviors through financial incentives and the risk of reducing evaluation to mechanisms of control and compliance. Although the quality component seeks greater alignment with SUS principles, there remains a need to scrutinize how these mechanisms relate to the development of capacities, the formative use of evaluation and the consideration of inequalities. The model once again reveals tensions between evaluation as a managerial instrument and as a practice aimed at strengthening PHC.

The fragility of programs aimed at institutionalizing evaluation stems from the unequal response capacity of subnational entities, the tendency toward the technocratization of evaluative processes, the limited capacity to use results, and the frequent political discontinuity and turnover in institutional leadership. These aspects highlight institutional vulnerability and reinforce the need to consolidate evaluation as a state policy through the development of evaluation policies focused on assessing policies and programs ^2^ ^,^ ^6^ ^,^ ^15^ ^,^ ^19^.

## Institutionalization of evaluation

Evaluation can improve the decisions of public managers by allowing them to identify strengths and weaknesses and to understand both the expected and unintended effects of policies and programs. This, in turn, strengthens governance capacity and enables better performance and a more rational use of resources ^20^. On the other hand, without an understanding of the changes that the use of evaluation may bring about, we will remain far from incorporating it as an integral part of the ethics, culture and responsibilities of work ^21^. Therefore, the term influence must be understood beyond the dimensions of approach, intentionality and temporality, incorporating the transformations that evaluation can generate in the thinking and actions of those involved, in the interactions among them, and in organizational practices and decisions ^6^ ^,^ ^20^ ^,^ ^22^ ^,^ ^23^. When analyzing M&E/PHC, its processual nature must be considered, given the complexity of the SUS and the strategic role of PHC. Institutionalizing evaluation requires a systemic approach that encompasses its multiple functional dimensions and their interrelationships ^24^.

In this context, the opening lecture by Jean-Louis Denis at the National Seminar on Institutionalization of Health Evaluation in 2010 (promoted by the Professor Fernando Figueira Institute of Integral Medicine - IMIP, acronym in Portuguese - and Abrasco) stands out, in which he synthesizes an institutional rationality whose elements can be adopted as “attributes of the institutionalization of evaluation” and compared with the events observed in M&E/PHC ^25^. Denis ^25^ warns that institutionalized evaluation may have different meanings for different sectors and actors within an organizational system, depending on the benefits the evaluations can offer and the possibilities for their qualified use. He outlines premises that justify the need to institutionalize evaluation: the perspective of transformation, given the growing complexity of policies and the need for more rational decisions; sustainability and mainstreaming, aimed at ensuring the permanence and central role of evaluation on the decision-making agenda; legitimacy, related to its actual influence on decisions; and the types of approach - objectives, theory and participation - which should be complementary and coordinated with institutional adequacy and the intentionality of results ^25^.

The author ^25^ identifies the main challenges for implementing the institutionalization and mainstream status of evaluation: overcoming its encapsulation within isolated structures; developing negotiation capacity to influence decision-making; shaping leadership committed to the continuity of resources and evaluative capacity; fostering communities of practice focused on learning and innovation; generating demand for evaluation based on decision-making needs; ensuring the presence of expertise; and creating forums for discussion. He concludes by presenting possible dilemmas, highlighting the potential of evaluations to challenge political and/or managerial decisions; the cultural change promoted by encouraging dialogue and the exposure of controversies; and the need to fund evaluation processes. As a precaution, he lists three consequences that may arise from institutionalization: political use, bureaucratic use and the paradoxical nature of evaluations ^25^.

This paradox relates to the coexistence of potentially contradictory expectations regarding institutionalized evaluation: while evaluation is expected to contribute to organizational learning, qualified decision-making and increased policy rationality, its incorporation into state structures may reduce its critical potential, making it an instrument of control, political legitimation or normative compliance ^25^. In the context of PHC in Brazil, this paradox is recurrent.

The tension Denis ^25^ also describes regarding the different meanings attributed to evaluation by various actors and sectors points to possible consequences of efforts to institutionalize PHC evaluation in Brazil. A first issue is the confrontation between conservative and innovative perspectives, as observed in 2006, when investments prioritized the Results-Based Management Program in Primary Care ^26^, a mechanism centered on targets and objectives that contrasted with the EQI’s efforts to strengthen evaluative capacity and train professionals and managers. Moreover, although the PMAQ repositioned evaluation as a strategic management instrument, its linkage to performance-based certification and incentive-based funding introduced a logic of meritocracy, reflecting tensions between different institutional rationalities.

Also noteworthy are the governance culture and leadership appointment practices that have historically permeated public administration in Brazil. Therefore, another point for reflection concerns the professional preparedness of institutions to implement these programs. How evaluative practices are understood and used varies across organizational levels, resulting in a multidimensional evaluative culture. The paradox described by Denis ^25^ tends to reproduce itself even when regulations are clear. A case in point is the ministerial ordinances that regulated the PNAB and defined the responsibilities related to M&E initiatives ^27^ ^,^ ^28^. Although they recommend that strategies for institutionalizing evaluation should be coordinated by state and federal managers, they do not establish consistent guidelines for structuring a policy, nor do they provide action plans, funding sources or sustainability mechanisms. Thus, evaluation is frequently used as an instrument of control and political exploitation, generating harmful initiatives such as the dismantling of the PMAQ and the implementation of Previne Brasil ^1^ ^,^ ^12^.

In our view, a Brazilian National Evaluation Policy for PHC, integrated with SUS principles, is essential, as it can serve as a pillar of democratic governance and contribute to the development of a Brazilian National Evaluation Policy for the SUS itself. This seems to be a natural requirement, given the central role of PHC in shaping Health Care Networks (RAS, acronym in Portuguese) and in organizing the SUS. In this sense, such a policy must be more solidly structured and less permeable to the factors that continue to threaten the structures of the SUS. These stem from different sources, many of which are politically driven and, in some cases, strategically embedded within the system’s institutional framework.

Some attributes should be considered regarding the extent to which a proposed evaluation policy is permeable to these influences. The first concerns the need for governance to be coordinated with the tripartite nature of the SUS, while also recognizing the specificities and demands inherent in the different contexts in which macro- and micro-level work processes occur. This reflects territorial and organizational particularities, as well as citizens’ expectations regarding policies and the need for timely responses to emerging issues. In this regard, the coordination of evaluative practices, supported by complementary theoretical frameworks, is crucial to enabling different actors to assess strategies and projects in alignment with the policy’s guidelines ^20^ ^,^ ^24^.

A second foundational attribute concerns how the meaning of evaluation is defined in the policy. Shared governance, the complexity of service organization and the principle of comprehensiveness require a clear definition of what it means to evaluate. This meaning should consider its theoretical grounding and the multiple values present at the various levels of complexity within the SUS, which influence and are influenced by organizational, political and social contexts. The formulation of this meaning must already signal the need to balance the dimensions of organizational learning and accountability ^29^.

A third attribute relates to the need for cooperation, which implies recognizing that interrelationships are as relevant as individual involvement. This perspective tends to foster greater commitment to decision making and strengthen evaluative practices. It should be noted that the involvement of external actors - through the formation of collaborative networks such as scientific and academic institutions and professional associations - is key to broadening the debate on evaluation outcomes, ethical standards, and theoretical and methodological frameworks ^20^.

A fourth relevant attribute is the recognition that the different levels of governance and actors involved in designing an evaluation policy operate within environments shaped by historically established beliefs, behaviors and values. Here, participatory approaches, or what is known as culturally responsive evaluation, gain importance, as they may promote the acculturation of evaluation, providing greater legitimacy and deeper embedding of its practices in territories ^20^ ^,^ ^29^ ^,^ ^30^ ^,^ ^31^.

Lastly, we highlight the need to monitor the degree of institutionalization that evaluation acquires over time. Such monitoring should consider both the breadth at the organizational level - encompassing the development of practices and the building of capacities - and the maturity of these practices, assessing dimensions such as regularity, credibility, legitimacy, transparency, availability of trained professionals, accountability and the dissemination of results. In addition, it should define the periodicity of monitoring and the degree of openness to updating action plans, allowing the policy to be adjusted in light of monitoring findings ^32^ ^,^ ^33^ ^,^ ^34^.

This final attribute points to the need to analyze the interface between the existence of an evaluation policy and the effective institutional capacity to generate and use its results, as discussed by Hudib & Cousins ^32^. The ecological framework proposed by the authors makes it possible to understand how different contexts shape the characteristics of an evaluation policy and suggests moderating variables, such as the role of the evaluator, interpersonal relationships, stakeholder engagement and organizational learning, which contribute to the interaction between these two capacities that are essential for an organization or system seeking to institutionalize evaluation ^32^ ^,^ ^33^ ^,^ ^34^ ^,^ ^35^.

The historical and analytical outline presented above reveals that the advances in the institutionalization of evaluation in PHC in Brazil have been marked by recurring tensions between different institutional rationalities, political and bureaucratic uses of evaluation, and weaknesses in the sustainable development of capacities. These limitations show that institutionalization is not achieved solely through the creation of normative instruments, information systems or incentive mechanisms; rather, it requires the explicit coordination of frameworks capable of reconnecting evaluation with the ethical, social and territorial purposes of the SUS.

## Health evaluation and the challenges of contemporary social transformations

The debate on the institutionalization of evaluation in PHC cannot dispense with a broader reflection on the population’s living and health conditions. The weaknesses already identified indicate the need for evaluative frameworks capable of expanding the meaning of evaluation beyond service performance or target attainment.

In this context, PHC plays a strategic role by assuming responsibility for reorienting the care model, anchored in the territory as a space where life, inequalities and institutional responses to health needs are produced. However, the concept of territory has often been used in a restricted sense, limited to its managerial dimension, which constrains the understanding of its analytical potential for capturing the interactions between social, environmental and health processes that shape the contemporary challenges of health.

There is a need to revisit the relationship of PHC and RAS with the organization of society and its effects on the environment and health. Two premises should guide this reflection, beginning with a closer engagement between public institutions and the population. The first can be summarized in the notion of “resilience”, here used to describe the everyday strategies of adaptation and coping developed by individuals and communities, generally in the short and medium term, in response to persistent adverse conditions imposed by the practical realities of daily life. These include, for example, those resulting from exposure to violence - stemming from a lack of employment and educational opportunities, drug trafficking and substance dependency, alcoholism, structural racism and sexism, and limited life prospects; from oppression - caused by undignified conditions of housing, sanitation, transportation, food and leisure; from climate change, natural disasters and the inaction of public authorities - such as prolonged droughts, fires and floods; from epidemiological events - arboviruses, zoonoses and obesity; from traffic accidents; and from the pressures of consumer society, which generate suffering and mental health disorders ^33^ ^,^ ^36^ ^,^ ^37^ ^,^ ^38^ ^,^ ^39^.

Resilience in the SUS involves the capacity for preparation, response, learning and institutional transformation in the face of different types of stressors, including chronic shocks and intentional political shocks ^40^. From this perspective, resilience is inseparable from political choices, governance models, funding and the system’s capacity to reorganize its structures and functions, thereby acquiring a political and strategic character ^40^. This approach broadens the meaning of health accountability and reinforces the need for evaluative processes capable of capturing not only adaptive responses within territories but also the institutional conditions that sustain or limit the SUS’s capacity to learn from crises and strengthen itself over time.

Another premise concerns “transformations”, which involve deeper and more enduring structural changes associated with rearrangements in modes of production, power relations and forms of social organization, with long-term impacts on territories. This occurs, for example, when a community is affected by economic development strategies such as the installation of industries and other productive activities, or when it is subjected to everyday oppression by militias and other types of criminal organizations - factors that may lead to behavioral and cultural changes and the emergence of new forms of social organization ^33^ ^,^ ^36^ ^,^ ^37^ ^,^ ^38^ ^,^ ^39^.

Such processes are not seen as occurring in isolation; rather, they often coexist and overlap in the concrete experiences of populations and health services. The distinction proposed here therefore aims to highlight different scales, time frames and social dynamics that affect living conditions, forms of collective organization and institutional responses within territories. These are real-life contexts of interaction between people and services, requiring a better understanding of the territory in order to guide interventions and their evaluation. At the same time, commitment and the ethics of responsibility should inform capacity-building efforts aimed at strengthening PHC through interventions aligned with social transformations ^34^ ^,^ ^41^ ^,^ ^42^ ^,^ ^43^.

This perspective - required of professionals and managers - stems from the transformations humanity has undergone as a consequence of its historical relationship with natural resources. The same attitude is therefore expected of researchers and evaluation professionals. Beyond theoretical and logical models, the focus should be on open theories of change that situate interventions within a broader context of transformation ^33^ ^,^ ^43^ “...*which means dealing with dynamics of complexity in a world characterized by turbulence, uncertainty, unpredictability and uncontrollability*” ^38^ (p. 29).

If social transformations require transformative interventions, evaluation must also confront this challenge. The following questions emerge as guiding principles: How can health evaluation be guided to align with the transformations required by interventions? How can health evaluation be guided to focus on people and the spaces they inhabit, beyond the interventions themselves, thereby contributing to equity and sustainability? The search for answers requires an expanded debate on possible directions, organized within a conceptual framework capable of supporting an evaluation policy ^36^ ^,^ ^43^ (Box 1).

**Box 1:** Challenges in developing a conceptual framework for primary health care (PHC) evaluation in Brazil.

GUIDING QUESTION How can health evaluation be guided so that it aligns with the transformations required by interventions and focuses on people and the spaces they inhabit, thereby contributing to the promotion of equity and sustainability?			
Strategy	Perspective	Characteristics	Possibilities
Mobilizing guiding perspectives to inform the formulation of a PHC evaluation policy in light of social transformations, with the aim of: - promoting the social legitimacy of evaluation; - expanding its meaning beyond bureaucratic control; - reconnecting it with the principles of equity, comprehensiveness and sustainability.	2030 Agenda ^44^ Food and nutrition security Inclusive and quality education Gender equality Environmental sustainability Social protection and equity Addressing environmental change	A systemic perspective grounded in the economic, social and environmental dimensions	An expanded understanding of territory, combined with the recognition of ethical, professional and social principles
	Brousselle & McDavid ^45^ ^,^ ^46^	Adoption of open theories Expansion of global mindset Incorporation of a long-term perspective Valuing the principles of sustainability and equity Conducting prospective evaluations Integration of different cultural perspectives Incorporation and coordination of multidisciplinary knowledge Updating ethical and professional standards and evaluation training processes	Greater coordination between interventions and the environmental and territorial contexts in which they are implemented Understanding of the exploitation of natural resources and its consequences Consideration of the time frames involved in the manifestation of environmental and socioeconomic impacts An integrated approach to the social, environmental and economic determinants of human health Critical systematization of accumulated knowledge Recognition and valuing of knowledge produced by local populations Continuous interdisciplinary collaboration Adoption of an expanded conception of territory, grounded in ethical principles and in the perspective of planetary health
DESIRED FUTURE STATE PHC evaluation as a strategic, ethical and dialogical practice, consistent with the principles of the SUS and with the contemporary challenges affecting territories			

SUS: Brazilian Unified National Health System.

Source: prepared by the authors.

Important guiding perspectives in this regard include the 2030 Agenda of the United Nations (UN) and the Sustainable Development Goals (SDGs). The Agenda, endorsed by 193 countries in 2015 - including Brazil - comprises 17 SDGs and should serve as a guiding framework for public officials in designing their initiatives. Its systemic vision, grounded in economic, social and environmental dimensions, makes it possible to shape an evaluation policy that brings stakeholders closer to a broader understanding of territory and to the valuation of ethical, professional and social principles ^44^.

The proposals of Brousselle & McDavid ^45^ may help illuminate this task. The authors suggest principles to guide evaluative practice and inform policy: the adoption of open theories, attuned to the connections between interventions and the environment in which they are implemented; a global mindset that enables links to be made between the exploitation of natural resources and its consequences; long-term thinking that recognizes the time required for environmental and socioeconomic impacts to become perceptible; the adoption of sustainability and equity as core values, understood as determinants of human health; the use of prospective evaluations employing theory-based approaches; the integration of diverse cultural perspectives, recognizing locally produced knowledge; the incorporation of multidisciplinary knowledge, bringing together insights from different fields; the adoption of new ethical and professional standards that incorporate broader conceptions of territory; the updating of assumptions in evaluation training processes, incorporating ethical considerations and the dimensions of planetary health; and the ethical defense of evaluation and the independence of evaluators, safeguarding democratic values and advancing equity ^44^. 

Considering these principles, the authors propose a framework to support an evaluative approach based on theories of change that link interventions to the dimensions of planetary health. This framework encompasses a human system (health, equity and prosperity) and a natural system (land, water, biodiversity and pollution), while also including contextual factors such as governance, power relations and other external elements. It connects the 17 SDGs with these dimensions and offers a systemic perspective on interventions ^45^.

Building on this theoretical proposition and seeking to apply it to the subject of this article, we argue that, when considering the SDGs, the guidelines of an evaluation policy should raise questions and approaches related to individuals, their social contexts, the characteristics of the natural environment and how communities organize themselves within a given space. Food security, inclusive education, gender equality, sustainable resource management, social protection and the mitigation of environmental degradation are SDGs whose indicators have direct implications for individual and public health within territories. This perspective should therefore be incorporated into M&E/PHC ^45^ ^,^ ^46^.

Another direction concerns participatory approaches and culturally responsive evaluation. Actively involving stakeholders in the design and implementation of evaluation incorporates knowledge produced from different perspectives, strengthening the value of and commitment to the process while enhancing its robustness and legitimacy. In addition, interdisciplinary connections should be fostered to support inclusive approaches aimed at the shared construction of meaning and the production of knowledge in contexts of complexity and transformation ^33^ ^,^ ^45^ ^,^ ^46^ ^,^ ^47^ ^,^ ^48^ ^,^ ^49^ ^,^ ^50^ ^,^ ^51^.

At present, M&E/PHC is guided by a restricted conception of evaluation. Although the redesign of federal funding incorporates components associated with bonding, follow-up and quality, its operationalization remains focused on performance indicators and financial incentive mechanisms. This situation needs to change so that evaluation can move beyond technocratic and fragmented approaches and consolidate itself as a strategic, ethical and dialogical practice.

This article has argued that the limits observed in institutionalization processes highlight the need for frameworks capable of expanding the meaning and social legitimacy of evaluation. The incorporation of approaches guided by a broader understanding of territory, by principles of equity and sustainability, and by participatory and culturally responsive processes offers pathways to reconnect evaluation with the principles of the SUS.

Rather than being limited to normative or operational arrangements, the institutionalization of evaluation should be grounded in decision-making processes, everyday practice and the relationships between the state and society, affirming evaluation as a constitutive element of the ethics of public service, democratic governance and social responsiveness - an ally of democracy and social justice, and a fundamental condition for sustainability and for strengthening the SUS itself.

## Data Availability

The sources of information used in the study are indicated in the body of the article.
