# Supplementary material for: Evaluation in primary health care in Brazil: reflections on institutionalization
Source: Cad Saude Publica. 2026 Nov 9;42(Suppl 2):e00182025. doi: 10.1590/0102-311XEN182025 (PMC13750642; doi:10.1590/0102-311XEN182025)
Supplement: Translation

# Translation: Avaliação na atenção primária à saúde no Brasil: reflexões sobre sua institucionalização

**DOI:** 10.1590/0102-311XPT182025

**Authors:** Eronildo Felisberto, Gabriella de Almeida Raschke Medeiros, Isabella Samico, Zulmira Maria de Araújo Hartz, Luciana Santos Dubeux, Ana Coelho de Albuquerque, Juliana Martins Barbosa da Silva Costa

**Affiliations:** 1 Grupo de Estudos em Gestão e Avaliação em Saúde, Instituto de Medicina Integral Prof. Fernando Figueira, Recife, Brasil.; 2 Secretaria de Atenção Primária à Saúde, Ministério da Saúde, Brasília, Brasil.; 3 Centro Acadêmico do Agreste, Universidade Federal de Pernambuco, Caruaru, Brasil.

**Keywords:** Avaliação em Saúde, Atenção Primária à Saúde, Objetivos de Desenvolvimento Sustentável, Institucionalização, Política de Saúde

## Abstract

O artigo apresenta reflexões sobre a institucionalização da avaliação em saúde. Ao sistematizar eventos ocorridos nos últimos 25 anos, no âmbito da avaliação na atenção primária à saúde (APS) no Brasil, contextualiza-os diante de abordagens teóricas buscando compreender processos de mudanças decorrentes dos movimentos que moldaram práticas e o uso da avaliação. Ao considerar que a institucionalização da avaliação exige uma abordagem sistêmica, destaca a influência dessas mudanças no pensamento, ações e interações dos envolvidos, nas práticas e decisões organizacionais. A avaliação na APS deve firmar-se como prática estratégica, ética e dialógica, alinhada aos compromissos democráticos do Sistema Único de Saúde (SUS) e aos desafios das transformações sociais contemporâneas. Inspirado em concepções teóricas sobre saúde planetária e avaliação transformadora, defende que a avaliação assuma intencionalmente um papel de transformação social e espaço de escuta qualificada, visibilidade das vulnerabilidades, promoção da equidade e articulação intersetorial. Sugere que sua institucionalização não se limite aos arranjos formais dos sistemas de saúde e considere estratégias de enraizamento nos territórios e nas relações entre Estado e sociedade. Pretende animar o debate ao propor pistas de como a institucionalização da avaliação na APS pode adquirir uma legitimidade mais ampliada e se consolidar como parte da ética do serviço público e da responsividade social.

Artigo publicado em 2024 ^1^ por pesquisadores da Rede de Pesquisa em APS da Associação Brasileira de Saúde Coletiva (Rede APS/ABRASCO), pavimentou o caminho para uma reflexão necessária sobre os rumos da avaliação na atenção primária à saúde (APS) no Brasil. O texto apresenta um histórico das iniciativas de monitoramento e avaliação da APS, propõe uma agenda de pesquisa comprometida com o fortalecimento da Estratégia Saúde da Família (ESF) e com a consolidação do Sistema Único de Saúde (SUS), e defende a construção de uma Política Nacional de Monitoramento e Avaliação para orientar a gestão e o cuidado da APS no SUS.

Para avançar nessa reflexão, este artigo tem como objetivo desenvolver uma análise histórico-crítica da institucionalização da avaliação na APS no Brasil, articulada a uma reflexão teórica sobre seus fundamentos, tensões e desafios, à luz das transformações sociais e territoriais contemporâneas. Adotou-se um recorte intencional, institucional e político de eventos relevantes que, nos últimos 25 anos, conformaram o cenário do Monitoramento e Avaliação da APS no Brasil (M&A/APS), tomando como base o modelo proposto pelo Ministério da Saúde no período 2003-2006, para a estruturação de uma política de avaliação para a APS ^2^.

## Trajetória e mecanismos de avaliação da APS no Brasil

Duas iniciativas importantes antecederam esse período. O Sistema de Informação da Atenção Básica (SIAB), criado em 1998 como instrumento gerencial para acompanhamento das ações e resultados das atividades das equipes da ESF, incorporou características como a micro espacialização dos dados, a geração de indicadores voltados ao ciclo de organização das ações e das informações por níveis de agregação que contribuíram com o debate sobre a responsabilidade sanitária ^3^. Nesse mesmo ano, o Pacto de Indicadores da Atenção Básica (PIAB) regulamentou o processo de acompanhamento e avaliação com base em indicadores, fluxos e prazos, influenciando o repasse financeiro aos municípios, definindo, também, o papel de cada instância gestora na negociação tripartite e o estabelecimento de compromissos à luz das Normas Operacionais Básicas ^4^.

Nos anos subsequentes, houve uma forte expansão da ESF e adensamento de sua centralidade na conformação da APS. Isto exigiu uma nova configuração institucional do Ministério da Saúde. Foram criados, nos anos 2000, o Departamento de Atenção Básica e a Coordenação de Acompanhamento e Avaliação. A valorização desse lócus institucional possibilitou o amadurecimento dos processos de M&A, fomentou a realização de pesquisas avaliativas e a articulação com instituições formadoras. Tais iniciativas buscaram qualificar profissionais e assegurar recursos financeiros para a avaliação da ESF no âmbito do Projeto de Expansão e Consolidação do Saúde da Família (PROESF) ^5^.

No período 2003-2006 se observou significativo avanço no debate em torno do M&A/APS, com estratégias voltadas para sua institucionalização. Processo impulsionado pela proposição de uma política setorial de avaliação, a partir de um debate ampliado no âmbito do Ministério da Saúde e da Comissão Intergestores Tripartite, que definiu algumas diretrizes: parcerias com instituições acadêmicas e associações profissionais; mobilização de atores institucionais; identificação de fontes de financiamento; definição de estratégias para qualificação profissional em avaliação ^2^ ^,^ ^6^ ^,^ ^7^.

Essas diretrizes nortearam estratégias articuladas visando à disseminação e ao fomento da cultura avaliativa no âmbito da APS: reformulação do SIAB e a disponibilização pública dos dados; dinamização do processo de negociação do PIAB; produção, análise e divulgação de indicadores de outros sistemas de informação; desenvolvimento de instrumentos facilitadores do uso das informações; fomento e gestão de pesquisas; publicização de experiências exitosas; qualificação profissional; busca de financiamento para avaliação ^2^ ^,^ ^6^.

Em decorrência, projetos inovadores buscaram superar as práticas avaliativas restritas às intervenções: Fortalecimento da Capacidade Técnica das Secretarias Estaduais de Saúde em M&A (2004), por meio de cooperação técnica com centros colaboradores; Estudos de Linha de Base do PROESF (2005), privilegiando instituições nacionais de pesquisa com acompanhamento independente pela Abrasco; Avaliação para a Melhoria da Qualidade (AMQ) (2005), instrumento de autoavaliação direcionado à ESF. Destaque-se o apoio institucional à criação do Grupo Técnico de Monitoramento e Avaliação de Programas e Políticas de Saúde (GT-Avaliação) da Abrasco, incentivando a cooperação entre instituições acadêmicas e os serviços de saúde ^8^ ^,^ ^9^ ^,^ ^10^ ^,^ ^11^ ^,^ ^12^. Iniciativas que compuseram a Política Nacional de Monitoramento e Avaliação da Atenção Básica (PNMA/AB), que atuou “...*como um padrão institucional para promover o desenvolvimento de uma cultura avaliativa, além de outros que orientaram a prática avaliativa na AB no Brasil…*” ^6^ (p. 38), como o Pacto pela Saúde (2004) e a Política Nacional da Atenção Básica (PNAB) (2006) ^6^ ^,^ ^9^ ^,^ ^12^.

Os marcos citados anteriormente definiram diretrizes e responsabilidades para o M&A/APS. Porém, os movimentos da gestão federal, no período 2007-2010, se restringiram a uma pesquisa amostral sobre a situação da APS no Brasil, ao apoio à Rede APS e às ações definidas para a segunda fase do PROESF. A partir de 2011 é que surgem novos eventos na direção da institucionalização da avaliação, com a instituição do Programa Nacional de Melhoria do Acesso e da Qualidade da Atenção Básica (PMAQ-AB) ^12^ ^,^ ^13^.

Esse programa incentivou a revisão do AMQ, resultando no documento *Autoavaliação para Melhoria do Acesso e da Qualidade da Atenção Básica - AMAQ* ^14^, que ampliou a possibilidade de aplicação dos padrões de qualidade para outros modelos de atenção, além da ESF. O PMAQ propôs uma maior responsabilização pública, melhoria contínua da qualidade e valorização do desempenho das equipes, incorporando mecanismos de avaliação, contratualização de metas e repasse financeiro vinculado ao desempenho, o que representou uma inflexão na política de financiamento e gestão da APS ^12^ ^,^ ^13^ ^,^ ^15^ ^,^ ^16^.

O PMAQ buscou integrar avaliação e financiamento por meio da articulação entre dimensões quantitativas e qualitativas do cuidado. Sua arquitetura contemplava: adesão voluntária dos municípios; contratualização de compromissos entre equipes e gestão; autoavaliação das equipes; monitoramento contínuo; avaliação externa; certificação por desempenho; incentivo financeiro. Isto reposicionou a avaliação como instrumento central da gestão da APS, incorporando-a como elemento indutor de transformações, e buscou ressaltar seu papel formativo e estratégico. A APS passou a contar com um processo contínuo de produção de dados para alimentar o planejamento local, fortalecendo a capacidade institucional ao estimular o uso da avaliação por gestores e profissionais em distintos níveis, além da incorporação da avaliação externa por instituições de pesquisa ^15^ ^,^ ^16^.

Em 2013, o Ministério da Saúde instituiu o sistema e-SUS Atenção Primária e o Prontuário Eletrônico do Cidadão. Essas iniciativas buscaram superar limitações do SIAB, permitindo tanto o registro de produção, quanto o acompanhamento longitudinal do cuidado. Ampliou-se o escopo de dados disponíveis, favorecendo a interoperabilidade entre sistemas, contribuindo de forma relevante com o M&A/APS ^1^ ^,^ ^13^ ^,^ ^15^ ^,^ ^16^.

Apesar desses avanços, observa-se, a partir de 2016, mais uma inflexão no cenário da APS. A adoção de diretrizes políticas contrapostas aos princípios do SUS, intensificaram movimentos institucionais de desmobilização e descrédito de iniciativas como o PMAQ. Em 2019, instituiu-se o Previne Brasil como modelo de financiamento da APS, que passou a vincular o repasse financeiro aos municípios a critérios como: capitação ponderada com base na população cadastrada, pagamento por desempenho de indicadores de produção e incentivo para ações estratégicas, desconsiderando o princípio constitucional do acesso universal à saúde ^1^ ^,^ ^17^.

Em 2024, com a retomada dos processos democráticos e de reordenação do cofinanciamento federal, o Ministério da Saúde readequou esses mecanismos aos princípios do SUS, instituindo um componente de qualidade com indicadores pactuados de forma tripartite ^18^. Na perspectiva da avaliação o novo modelo recoloca desafios já observados na experiência do PMAQ, especialmente quanto à centralidade dos indicadores, a indução de comportamentos por incentivos financeiros e ao risco de redução da avaliação a mecanismos de controle e conformidade. Ainda que o componente de qualidade busque maior alinhamento com os princípios do SUS, persiste a necessidade de problematizar como esses dispositivos se articulam à construção de capacidades, ao uso formativo da avaliação e à consideração das desigualdades. O modelo revela, mais uma vez, as tensões entre avaliação como instrumento gerencial e como prática voltada ao fortalecimento da APS.

A fragilidade dos programas voltados à institucionalização da avaliação decorre da desigual capacidade de resposta dos entes subnacionais, da tendência à tecnocratização dos processos avaliativos, da baixa qualificação para o uso dos resultados e da frequente descontinuidade política e das lideranças institucionais. Tais aspectos evidenciam a vulnerabilidade institucional e reforçam a necessidade de consolidar a avaliação como política de Estado, construindo políticas de avaliação voltadas à avaliação de políticas e programas ^2^ ^,^ ^6^ ^,^ ^15^ ^,^ ^19^.

## Institucionalização da avaliação

A avaliação pode aprimorar as decisões dos gestores públicos ao permitir a identificação de pontos fortes e fracos e a compreensão dos efeitos esperados e os não intencionais das políticas e programas, fortalecendo a capacidade de governança e possibilitando melhor desempenho e racionalização dos recursos ^20^. Por outro lado, sem uma compreensão sobre as mudanças que o uso da avaliação pode causar, continuaremos distantes de incorporá-la como parte integrante da ética, da cultura, e das responsabilidades do trabalho ^21^. Deve-se, portanto, ampliar o entendimento do termo influência, para além das dimensões: abordagem, intencionalidade e temporalidade, incorporando as transformações que a avaliação pode proporcionar no pensamento e nas ações dos envolvidos, nas interações entre eles e nas práticas e decisões organizacionais ^6^ ^,^ ^20^ ^,^ ^22^ ^,^ ^23^. Ao analisar o M&A/APS, deve-se considerar seu caráter processual, dada a complexidade do SUS e o papel estratégico da APS. A institucionalização da avaliação exige uma abordagem sistêmica que contemple suas múltiplas dimensões funcionais e suas inter-relações ^24^.

Nesse cenário, destaca-se a conferência de abertura de Jean-Louis Denis, no Seminário Nacional sobre Institucionalização da Avaliação em Saúde em 2010 (promovido pelo Instituto de Medicina Integral Prof. Fernando Figueira - IMIP e pela Abrasco), na qual o autor sintetiza uma racionalidade institucional, cujos elementos podem ser adotados como “atributos da institucionalização da avaliação” e confrontados com os eventos ocorridos no M&A/APS ^25^. Denis ^25^ alerta para diferentes significados que a avaliação institucionalizada assume para diferentes setores e atores de um sistema organizacional, a depender dos benefícios que as avaliações podem oferecer e das possibilidades de seu uso qualificado. Enumera premissas que justificam a necessidade de institucionalizar a avaliação: a perspectiva de transformação, diante da crescente complexidade das políticas e da necessidade de decisões mais racionais; a sustentabilidade e o mainstream, visando à permanência e centralidade da avaliação na agenda decisória; a legitimidade, relacionada à sua real influência sobre as decisões; os tipos de abordagem - objetivos, teoria e participação, que devem ser complementares, articuladas à adequação institucional e à intencionalidade dos resultados ^25^.

O autor ^25^ identifica os principais desafios para implementar a institucionalização e o status de mainstream da avaliação: superar o encapsulamento em estruturas isoladas; desenvolver a capacidade de negociação para influenciar na tomada de decisão; formar lideranças comprometidas com a perenidade dos recursos e capacidade avaliativa; fomentar comunidades de práticas de aprendizagem e inovação; induzir demandas por avaliação baseadas nas necessidades do processo decisório; assegurar a presença de expertises; oportunizar fóruns de discussão. Conclui apresentando possíveis dilemas, ressaltando: o caráter potencialmente contestatório das avaliações frente a decisões políticas e/ou gerenciais; a mudança cultural promovida pelo estímulo ao diálogo e à exposição de controvérsias; e a necessidade de financiamento para os processos avaliativos. Como precaução, enumera três consequências que podem decorrer da institucionalização: o uso político; o uso burocrático; e o caráter paradoxal das avaliações ^25^.

Esse paradoxo refere-se à coexistência de expectativas potencialmente contraditórias em relação à avaliação institucionalizada: ao mesmo tempo em que se espera que a avaliação contribua para o aprendizado organizacional, a qualificação das decisões e a ampliação da racionalidade das políticas, sua incorporação às estruturas do Estado pode reduzir seu potencial crítico, transformando-a em instrumento de controle, legitimação política ou conformidade normativa ^24^. No contexto da APS no Brasil, esse paradoxo manifesta-se de forma recorrente.

A tensão também descrita por Denis ^25^ sobre diferentes significados para distintos atores e setores, permite apontar possíveis consequências das iniciativas de institucionalização da avaliação desenvolvidas na APS no Brasil. Um primeiro ponto é o confronto entre perspectivas conservadoras e inovadoras, como observado em 2006, quando os investimentos priorizaram o Programa de Gestão por Resultados na Atenção Básica ^26^, dispositivo centrado em metas e objetivos e que contrastava com os esforços do AMQ de fortalecimento da capacidade avaliativa e formação de profissionais e gestores. Também, não obstante o reposicionamento que o PMAQ proporcionou à avaliação como instrumento estratégico da gestão, sua vinculação à certificação por desempenho e ao financiamento por incentivo introduziu uma lógica de meritocracia, refletindo tensões entre diferentes racionalidades institucionais.

Deve-se considerar, ainda, a cultura de governança e a lógica de ocupação de cargos diretivos que historicamente permeiam a gestão pública no Brasil. Portanto, outro ponto a ser refletido é sobre o preparo profissional das instituições para dar conta dos programas. A apreensão sobre as práticas avaliativas e o uso da avaliação variam entre os diferentes níveis organizacionais, traduzindo-se numa cultura avaliativa multidimensional. O paradoxo descrito por Denis ^25^ tende a se reproduzir, mesmo diante de normativas claras. Como exemplo, temos as portarias que regulamentaram a PNAB e definiram as atribuições das ações de M&A ^27^ ^,^ ^28^. Embora recomendem que estratégias de institucionalização da avaliação deverão ser articuladas pelos gestores estaduais e federal, não estabelecem diretrizes consistentes para estruturação de uma política, tampouco preveem planos de ação, fontes de financiamento ou mecanismos de sustentabilidade. Constata-se, portanto, o uso corrente da avaliação como instrumento de controle e seu uso político, gerando iniciativas deletérias como a desmobilização do PMAQ e a implementação do Previne Brasil ^1^ ^,^ ^12^.

Compreendemos como basal a existência de uma Política Nacional de Avaliação para a APS integrada aos princípios do SUS, que se constitua como um pilar da governança democrática e contribua para estruturação de uma Política Nacional de Avaliação do próprio SUS. Isto nos parece uma exigência natural, dada a centralidade da APS na conformação das Redes de Atenção à Saúde (RAS) e na organização do SUS. Nesse sentido, tal política precisa adquirir contornos mais sólidos e menos permeáveis aos fatores que insistem em ameaçar as estruturas do SUS, que provêm de vetores diversos, muitos dos quais estão politicamente posicionados, e em alguns casos, estrategicamente instalados no arcabouço do sistema.

Alguns atributos devem ser considerados no que tange ao grau de permeabilidade de uma política de avaliação a ser proposta. O primeiro deles diz respeito à necessidade de uma governança articulada ao caráter tripartite do SUS, mas que também reconheça as especificidades e demandas inerentes aos diferentes contextos em que ocorrem os macro e micro processos de trabalho. Isso decorre da existência de peculiaridades territoriais e organizacionais, bem como das expectativas dos cidadãos em relação às políticas e à necessidade de respostas oportunas a questões emergentes. Aqui, a articulação entre as práticas avaliativas, sustentada por referenciais teóricos complementares, mostra-se crucial para que os diversos atores possam avaliar estratégias e projetos em consonância com as diretrizes da política ^20^ ^,^ ^24^.

Um segundo atributo basilar é a definição do significado da avaliação a ser explicitado na política. A gestão compartilhada, a complexidade de organização dos serviços e o princípio da integralidade demandam uma definição clara sobre o que significa avaliar. Esse significado deve considerar o seu embasamento teórico e a pluralidade de valores circulantes nos diversos níveis de complexidade do SUS, que influenciam e são influenciados pelos contextos organizacionais, políticos e sociais. É essencial que o enunciado desse significado já sinalize para a ponderação entre as dimensões de aprendizagem organizacional e de *accountability* ^29^.

O terceiro refere-se à necessidade de cooperação, o que implica reconhecer que as inter-relações são tão relevantes quanto o envolvimento individual. Essa perspectiva tende a gerar maior comprometimento com a tomada de decisão e qualificar as práticas avaliativas. Ressalta-se que o envolvimento de atores externos, por meio da formação de redes colaborativas - instituições científicas, acadêmicas, associações profissionais -, é crucial para ampliar o debate sobre os resultados das avaliações, os padrões éticos e os referenciais teórico-metodológicos ^20^.

Um quarto atributo relevante é o reconhecimento de que os diferentes níveis de governança e atores envolvidos no desenho da política de avaliação estão inseridos em ambientes carregados de crenças, comportamentos e valores historicamente construídos. Aqui, ganham importância as abordagens participativas ou a chamada avaliação culturalmente responsiva que pode favorecer o processo de aculturamento da avaliação, promovendo maior legitimidade e enraizamento das suas práticas nos territórios ^20^ ^,^ ^29^ ^,^ ^30^ ^,^ ^31^.

Por último, destacamos a necessidade de monitorar o grau de institucionalização que a avaliação vai adquirindo ao longo do tempo. Tal monitoramento deve considerar tanto a amplitude no nível organizacional, que envolve desenvolvimento de práticas e construção de capacidades, quanto o grau de maturidade dessas práticas, avaliando dimensões como regularidade, credibilidade, legitimidade, transparência, disponibilidade de profissionais capacitados, responsabilização e disseminação de resultados. Além disso, deve contemplar a definição da sua periodicidade e do grau de abertura à atualização dos planos de ação, permitindo a adequação da política aos achados do monitoramento ^32^ ^,^ ^33^ ^,^ ^34^.

Esse último atributo remete à necessidade de analisar a interface entre a existência de uma política de avaliação e a efetiva capacidade institucional de produzir e utilizar seus resultados, conforme discutido por Hudib & Cousins ^32^. A estrutura ecológica proposta pelos autores possibilita compreender como diversos contextos moldam as características de uma política de avaliação e sugere variáveis moderadoras: papel do avaliador; relações interpessoais; engajamento das partes; e aprendizagem organizacional, que contribuem para a interação entre essas duas capacidades essenciais a uma organização ou sistema que pretenda institucionalizar a avaliação ^32^ ^,^ ^33^ ^,^ ^34^ ^,^ ^35^.

O percurso histórico e analítico apresentado, revela que os avanços na institucionalização da avaliação na APS no Brasil têm sido marcados por tensões recorrentes entre diferentes racionalidades institucionais, usos políticos e burocráticos da avaliação e fragilidades na construção sustentável de capacidades. Esses limites evidenciam que a institucionalização não se esgota na criação de dispositivos normativos, sistemas de informação ou mecanismos de incentivo, exigindo a explicitação de referenciais capazes de reconectar a avaliação às finalidades éticas, sociais e territoriais do SUS.

## Avaliação em saúde e os desafios das transformações sociais do nosso tempo

O debate sobre institucionalização da avaliação na APS não pode prescindir de uma reflexão mais ampla sobre as condições de vida e saúde da população. As fragilidades já apontadas indicam a necessidade de referenciais avaliativos capazes de ampliar o sentido da avaliação para além do desempenho de serviços ou do cumprimento de metas.

Nesse contexto, a APS ocupa uma posição estratégica ao assumir a responsabilidade pela reorientação do modelo de atenção, ancorada no território como espaço de produção da vida, das desigualdades e das respostas institucionais às necessidades de saúde. Contudo, é recorrente o uso do conceito de território de forma restrita à sua dimensão gerencial, o que limita a compreensão de sua potência analítica para apreender as interações entre processos sociais, ambientais e sanitários que moldam os desafios contemporâneos da saúde.

É necessário revisitar a relação da APS e das RAS com a organização da sociedade e os efeitos que essa organização produz sobre o meio ambiente e a saúde. Duas premissas devem orientar essa reflexão, partindo de uma efetiva aproximação das estruturas públicas com a população. A primeira pode ser sintetizada na noção de resiliência, aqui utilizada para descrever estratégias cotidianas de adaptação e enfrentamento desenvolvidas por pessoas e comunidades, geralmente situadas no curto e médio prazo, diante de condições adversas persistentes, impostas pelas questões práticas da vida cotidiana. Como exemplo, podemos citar aquelas decorrentes da exposição à violência - consequente à falta de oportunidades de emprego e educação, do tráfico de drogas e de sua dependência, do alcoolismo, do racismo e do machismo estrutural, da falta de perspectivas -, à opressão - causada pelas condições indignas de moradia, saneamento básico, transporte, alimentação, lazer -, às alterações climáticas, desastres naturais e decorrentes da inação dos agentes públicos - secas prolongadas, incêndios, enchentes -, aos eventos epidemiológicos - arboviroses, zoonoses, obesidade - acidentes de trânsito etc., às pressões da sociedade de consumo - geradoras de sofrimento e transtornos mentais ^33^ ^,^ ^36^ ^,^ ^37^ ^,^ ^38^ ^,^ ^39^.

A resiliência no SUS envolve a capacidade de preparação, resposta, aprendizado e transformação institucional diante de diferentes tipos de estressores, incluindo choques crônicos e choques políticos intencionais ^40^. Nessa perspectiva, a resiliência é indissociável das escolhas políticas, dos modelos de governança, do financiamento e da capacidade do sistema de reorganizar suas estruturas e funções, assumindo caráter político e estratégico ^40^. Essa abordagem amplia o sentido da responsabilização sanitária e reforça a necessidade de processos avaliativos capazes de apreender não apenas respostas adaptativas no território, mas também as condições institucionais que sustentam ou limitam a capacidade do SUS de aprender com crises e se fortalecer ao longo do tempo.

Uma outra premissa a ser observada são as transformações, que envolvem mudanças estruturais mais profundas e duradouras, associadas a rearranjos nos modos de produção, nas relações de poder e nas formas de organização social. Elas têm impactos de longo prazo sobre os territórios, como quando uma comunidade é afetada por estratégias de desenvolvimento econômico como instalação de indústrias e outros meios de produção; e até mesmo quando são vítimas de opressão cotidiana permanente por parte de milícias e outros tipos de organizações criminosas, fatores que podem levar a mudanças comportamentais e culturais e estabelecer novos modelos de organização social ^33^ ^,^ ^36^ ^,^ ^37^ ^,^ ^38^ ^,^ ^39^.

Reconhece-se que tais processos não se apresentam de forma estanque na realidade social, frequentemente coexistindo e se sobrepondo nas experiências concretas das populações e dos serviços de saúde. A separação proposta tem, portanto, a finalidade de evidenciar diferentes escalas, temporalidades e dinâmicas sociais que incidem sobre as condições de vida, os modos de organização coletiva e as respostas institucionais no território. São cenários reais nos quais se dá a interação entre as pessoas e os serviços, exigindo uma melhor compreensão sobre o território para delinear as intervenções e a sua avaliação. Por outro lado, o comprometimento e a ética da responsabilidade devem permear o processo de construção das capacidades profissionais, visando à qualificação da APS a partir de intervenções alinhadas com as transformações sociais ^34^ ^,^ ^41^ ^,^ ^42^ ^,^ ^43^.

Esse posicionamento, exigido dos profissionais e gestores, decorre dessas transformações pelas quais a humanidade vem passando, consequente a sua relação histórica com os recursos naturais. Espera-se, portanto, que essa mesma postura seja assumida por pesquisadores e profissionais da avaliação. Para além dos modelos teóricos e lógicos, deve-se focar em teorias de mudança abertas que coloquem a intervenção em um contexto mais amplo, de transformação ^33^ ^,^ ^43^ “...*o que significa lidar com dinâmicas de complexidade em um mundo caracterizado por turbulência, incerteza, imprevisibilidade e incontrolabilidade*” ^38^ (p. 29).

Se as transformações sociais exigem intervenções transformadoras, também a avaliação deve enfrentar esse desafio. As seguintes questões emergem como norteadoras: Como orientar a avaliação em saúde de modo que ela esteja alinhada às transformações necessárias às intervenções? Como orientar a avaliação em saúde a focar nos seres humanos e no espaço que ocupam, para além das intervenções per si, contribuindo com a equidade e a sustentabilidade? Na busca por respostas, é necessário um debate amplo sobre possíveis pistas e organizá-las em uma estrutura conceitual que dê sustentação a uma política de avaliação ^36^ ^,^ ^43^ (Quadro 1).

**Quadro 1:** Desafios à construção de uma estrutura conceitual para a avaliação da atenção primária à saúde (APS) no Brasil.

QUESTÃO NORTEADORA Como orientar a avaliação em saúde para que esteja alinhada às transformações necessárias às intervenções e focada nos seres humanos e no espaço que ocupam, contribuindo para a promoção da equidade e da sustentabilidade?			
Estratégia	Referencial	Características	Possibilidades
Mobilizar referenciais para a formulação de uma política de avaliação para a APS, à luz das transformações sociais, buscando: - legitimação social da avaliação; - ampliação do sentido para além do controle burocrático; - reconexão com os princípios de equidade, integralidade e sustentabilidade.	Agenda 2030 ^44^ Segurança alimentar e nutricional Educação inclusiva e de qualidade Igualdade de gênero Sustentabilidade ambiental Proteção social e equidade Enfrentamento das mudanças ambientais	Visão sistêmica pautada nas dimensões econômica, social e ambiental	Compreensão ampliada do território, associada à valoração de princípios éticos, profissionais e sociais
	Brousselle & McDavid ^45^ ^,^ ^46^	Adoção de teorias abertas Ampliação do pensamento global Incorporação de uma perspectiva de longo prazo Valorização dos princípios da sustentabilidade e da equidade Realização de avaliações prospectivas Integração de diferentes perspectivas culturais Incorporação e articulação de saberes multidisciplinares Atualização de padrões éticos e profissionais e dos processos de formação em avaliação	Maior articulação entre as intervenções e os contextos ambientais e territoriais de sua implementação Compreensão sobre a agressão aos recursos naturais e suas consequências Consideração das temporalidades envolvidas na manifestação dos impactos ambientais e socioeconômicos Abordagem integrada dos determinantes sociais, ambientais e econômicos da saúde humana Sistematização crítica do conhecimento acumulado Reconhecimento e valorização dos saberes produzidos pelas populações locais Colaboração interdisciplinar contínua Adoção de uma concepção ampliada de território, ancorada em princípios éticos e na perspectiva da saúde planetária
IMAGEM OBJETIVO Avaliação na APS como uma prática estratégica, ética e dialógica, coerente com os princípios do SUS e com os desafios contemporâneos que atravessam os territórios			

SUS: Sistema Único de Saúde.

Fonte: elaboração própria.

É nesse horizonte que se situam referenciais como a Agenda 2030 da Organização das Nações Unidas (ONU) e os Objetivos de Desenvolvimento Sustentável (ODS). A Agenda, subscrita por 193 países em 2015, entre eles o Brasil, é composta por 17 ODS e deve ser utilizada pelos agentes públicos como instrumento orientador de suas ações. Sua visão sistêmica pautada nas dimensões econômica, social e ambiental possibilita moldar uma política de avaliação que aproxime as partes interessadas da compreensão ampliada de território e da valoração de princípios éticos, profissionais e sociais ^44^.

As sugestões de Brousselle & McDavid ^45^ podem iluminar essa tarefa. Os autores propõem princípios que devem permear a prática avaliativa e delinear as políticas: adoção de teorias abertas, sintonizadas na conexão das intervenções com o ambiente de sua implementação; pensamento global, que permita vincular a agressão aos recursos naturais e suas consequências; pensamento a longo prazo, compreendendo o tempo que os impactos ambientais e socioeconômicos levam para ser perceptíveis; adoção de valores de sustentabilidade e equidade, compreendendo-os como determinantes da saúde humana; realização de avaliações prospectivas, utilizando abordagens baseadas em teorias; integração de diferentes perspectivas culturais, reconhecendo o conhecimento produzido localmente; incorporação de saberes multidisciplinares, agregando o conhecimento de diferentes áreas; adoção de novos padrões éticos e profissionais, que incorporem perspectivas de concepção ampliada de território; atualização dos pressupostos nos processos de formação em avaliação, incorporando a valoração ética e as dimensões da saúde planetária; defesa ética da avaliação e da independência dos avaliadores, resguardando os valores democráticos e na direção da equidade ^45^.

Considerando esses princípios, esses autores propuseram um *framework* de sustentação a uma abordagem avaliativa, baseada em teorias de mudança, relacionando as intervenções com as dimensões da saúde planetária, considerando um sistema humano (saúde, equidade e prosperidade); e um sistema natural (terra, água, biodiversidade e poluição). Sinaliza fatores contextuais como governança, relações de poder e outros de ordem externa. Essa abordagem conecta os 17 ODS com essas dimensões e oferece uma visão sistêmica das intervenções ^45^.

Tomando como base essa proposição teórica e, na tentativa de fazer uma translação para o objeto deste artigo, consideramos que as diretrizes de uma política de avaliação devem, ao considerar os ODS, suscitar perguntas e abordagens conectadas com o indivíduo, com sua inserção social, com as características naturais e de como a coletividade se organiza em determinado espaço. Segurança alimentar, educação inclusiva, igualdade de gênero, gestão sustentável dos recursos, segurança social, mitigação das agressões ao meio ambiente, são ODS cujos indicadores têm interface direta com a saúde individual e coletiva nos territórios. Essa perspectiva deve ser incorporada ao M&A/APS ^45^ ^,^ ^46^.

Outra pista a ser considerada consiste na perspectiva participativa e na avaliação culturalmente responsiva. Ao envolver ativamente os diversos atores interessados, no desenho e na execução da avaliação, insere-se o conhecimento construído sob diferentes concepções, o que fortalece o valor e o compromisso com o processo e promove maior robustez e legitimidade. Ainda, deve-se promover conexões entre diferentes disciplinas que podem apoiar abordagens inclusivas, visando à construção compartilhada de sentidos e à produção de conhecimento em cenários de complexidade e transformação ^33^ ^,^ ^45^ ^,^ ^46^ ^,^ ^47^ ^,^ ^48^ ^,^ ^49^ ^,^ ^50^ ^,^ ^51^.

Atualmente, o M&A/APS é norteado por uma concepção restrita de avaliação. Embora o redesenho do financiamento federal incorpore componentes associados ao vínculo, ao acompanhamento e à qualidade, sua operacionalização permanece centrada em indicadores de desempenho e mecanismos de indução financeira. Esse cenário necessita ser modificado, possibilitando que a avaliação transcenda as abordagens tecnocráticas e fragmentadas, consolidando-se como uma prática estratégica, ética e dialógica.

Este artigo argumentou que os limites observados nos processos de institucionalização evidenciam a necessidade de referenciais que ampliem o sentido e a legitimidade social da avaliação. A incorporação de abordagens orientadas por uma compreensão ampliada de território, pela equidade e sustentabilidade, e por processos participativos e culturalmente responsivos, oferece caminhos para reconectar a avaliação aos princípios do SUS.

A institucionalização da avaliação não deve restringir-se a arranjos normativos ou operacionais, mas buscar enraizamento nos processos decisórios, nas práticas cotidianas e nas relações entre Estado e sociedade, afirmando a avaliação como parte constitutiva da ética do serviço público, da governança democrática e da responsividade social - uma aliada da democracia e da justiça social e condição fundamental para sustentabilidade e para o fortalecimento do próprio SUS.

## Data Availability

As fontes de informação utilizadas no estudo estão indicadas no corpo do artigo.
